# Bilateral Tactile Feedback-Enabled Training for Stroke Survivors Using Microsoft Kinect^TM^

**DOI:** 10.3390/s19163474

**Published:** 2019-08-08

**Authors:** Abbas Orand, Eren Erdal Aksoy, Hiroyuki Miyasaka, Carolyn Weeks Levy, Xin Zhang, Carlo Menon

**Affiliations:** 1 Department of Intelligent Systems and Digital Design, School of Information Technology, Halmstad University, Spetsvinkelgatan 29, 30250 Halmstad, Sweden; 2 Department of Rehabilitation, Fujita Health University Nanakuri Memorial Hospital, 424-1 Oodori-cho, Tsu, Mie 514-1296, Japan; 3 Schools of Mechatronics Systems Engineering and Engineering Science, Simon Fraser University, 250-13450 102 Avenue, Surrey, BC V3T 0A3, Canada

**Keywords:** Kinect, stroke rehabilitation, bilateral training, tactile feedback

## Abstract

Rehabilitation and mobility training of post-stroke patients is crucial for their functional recovery. While traditional methods can still help patients, new rehabilitation and mobility training methods are necessary to facilitate better recovery at lower costs. In this work, our objective was to design and develop a rehabilitation training system targeting the functional recovery of post-stroke users with high efficiency. To accomplish this goal, we applied a bilateral training method, which proved to be effective in enhancing motor recovery using tactile feedback for the training. One participant with hemiparesis underwent six weeks of training. Two protocols, “contralateral arm matching” and “both arms moving together”, were carried out by the participant. Each of the protocols consisted of “shoulder abduction” and “shoulder flexion” at angles close to 30 and 60 degrees. The participant carried out 15 repetitions at each angle for each task. For example, in the “contralateral arm matching” protocol, the unaffected arm of the participant was set to an angle close to 30 degrees. He was then requested to keep the unaffected arm at the specified angle while trying to match the position with the affected arm. Whenever the two arms matched, a vibration was given on both brachialis muscles. For the “both arms moving together” protocol, the two arms were first set approximately to an angle of either 30 or 60 degrees. The participant was asked to return both arms to a relaxed position before moving both arms back to the remembered specified angle. The arm that was slower in moving to the specified angle received a vibration. We performed clinical assessments before, midway through, and after the training period using a Fugl-Meyer assessment (FMA), a Wolf motor function test (WMFT), and a proprioceptive assessment. For the assessments, two ipsilateral and contralateral arm matching tasks, each consisting of three movements (shoulder abduction, shoulder flexion, and elbow flexion), were used. Movements were performed at two angles, 30 and 60 degrees. For both tasks, the same procedure was used. For example, in the case of the ipsilateral arm matching task, an experimenter positioned the affected arm of the participant at 30 degrees of shoulder abduction. The participant was requested to keep the arm in that position for ~5 s before returning to a relaxed initial position. Then, after another ~5-s delay, the participant moved the affected arm back to the remembered position. An experimenter measured this shoulder abduction angle manually using a goniometer. The same procedure was repeated for the 60 degree angle and for the other two movements. We applied a low-cost Kinect to extract the participant’s body joint position data. Tactile feedback was given based on the arm position detected by the Kinect sensor. By using a Kinect sensor, we demonstrated the feasibility of the system for the training of a post-stroke user. The proposed system can further be employed for self-training of patients at home. The results of the FMA, WMFT, and goniometer angle measurements showed improvements in several tasks, suggesting a positive effect of the training system and its feasibility for further application for stroke survivors’ rehabilitation.

## 1. Introduction

A stroke is caused by interruptions in the blood supply to the brain [1]. It is serious and disabling [2] and is the cause of adult disability in many countries [2,3]. The hemiparesis of the contralateral upper limb is the most common deficit after stroke and affects more than 80% of stroke survivors [4]. Upper extremity (UE) impairments affect the activities of daily living of stroke survivors by causing disability in common activities such as reaching [5,6]. Meaningful hand activity six months post-stroke can be minimal [6], and long-term health effects continue to affect the quality of life of the patient. Therefore, improved interventions for the rehabilitation of stroke survivors are essential. However, traditional rehabilitation interventions result in continued impairment of stroke survivors [7,8]. In order to improve rehabilitation protocols and results, new approaches such as bilateral training, feedback application, and low-cost 3D motion capture systems such as Kinect^TM^ (Kinect is manufactured by Microsoft and is a motion sensing device) were investigated in this study.

### 1.1. Bilateral Training

Studies have shown that unaffected and affected limbs that were simultaneously moved in a bilateral training protocol were effective in the recovery of motor function in hemiplegic patients [9,10,11,12]. Activation of the nervous system and stimulation of the cerebral hemisphere by bimanual training were shown in another study [13]. Another study showed that contralateral movement was controlled by 90% of the nerve fibers in the cerebral cortex and the spinal cord, while the remaining 10% controlled ipsilateral movement [14]. Activities of daily living (ADLs) also require the use of both hands, making bilateral rehabilitation training necessary [15,16]. In a structured review and meta-analysis of studies made on bilateral movement training and stroke recovery, Cauraugh et al. [17] identified 48 potential research studies, of which 25 were compared to determine the cumulative effect of bilateral arm training on motor capabilities post-stroke. They concluded that pure bilateral or active and/or passive movement training protocols do not contribute to significant motor recovery. The applications of bimanual training with robots [18,19,20] and rhythmic auditory cueing [21] were investigated in the rehabilitation of stroke survivors. The possibility of the concept of bimanual training and companied devices in extending contemporary therapies is accepted. The combination of bimanual training with rhythmic auditory cueing was found to be partially efficient in the rehabilitation of chronic stroke patients.

### 1.2. Tactile Feedback

Afferent nerves carry tactile messages such as vibration, pressure, and stretch to the brain [22,23]. Shull et al. [24] defined haptics as the sense of touch, which includes vibration. They classified vibration feedback in the partial impairment category, which requires sensory augmentation. Haptic wearables were shown to be useful for partial impairment by facilitating motor control and rehabilitation [25], and a haptic wearables application was even beneficial for individuals with intact sensory information for behavioral deficit correction [24]. In a study conducted by Stepp and Matsuoka [26] on the differences between stimulation sites during vibrotactile feedback, they found that the location of skin stimulation was of less importance than other factors, such as learning rates. Vibrotactile feedback was applied for proprioception feedback [27] and in stroke subjects [28]. The sensory feedback was found to be directly correlated with accuracy and to be beneficial for increasing subject confidence [28]. Held et al. [28] found vibrotactile feedback to be more acceptable to stroke patients. They concluded that it can be used as a telerehabilitation device for training.

### 1.3. Kinect Application

Microsoft’s Kinect is a 3D motion capture system [29]. It can identify 25 body joint centers at a frequency of 30 Hz using a personal computer [30]. Considering Kinect’s great properties, such as portability, low cost, and its markerless design, it can easily be used for measuring human movement [30]. The validity of Kinect in a clinical study of people with Parkinson’s disease yielded a good interclass correlation coefficient, >0.9, and a Pearson’s *r* > 0.9 [31]. Webster et al. [32] carried out a systematic review of Kinect applications in the elderly for stroke rehabilitation. They concluded that the Kinect has great potential in providing therapy for such individuals. The Kinect was also applied for building financially accessible and medically beneficial systems for patient use [32]. The Kinect has been used for a virtual reality rehabilitation system [33], a virtual exercise guide and game-based rehabilitation system [34,35,36], an interactive visuotactile 3D virtual environment [37], and a brain-controlled interface-driven robotic upper limb exoskeleton [38].

### 1.4. Proprioception Training

Proprioception is the sense of body position [39]. It is the ability of the central nervous system to determine body part positioning in time [40] and space [41]. Proprioception is important for sensorimotor control and is required for muscle stiffness regulation, joint stability and speed, coordination, and balance [39,41,42,43]. Therefore, intact proprioception is essential for movement control and functioning [44]. Proprioception training has been shown to decrease the incidence of injuries and to improve proprioception accuracy in trained healthy subjects compared to nontrained healthy subjects [45,46,47,48]. A proprioception deficit is common among stroke patients. A recent research study [41] reported that up to 85% of stroke patients’ proprioception was affected after stroke. For those post-stroke patients with affected UE, the proprioception deficit was reported to be 30% to 48% [43]. Therefore, interventions for proprioceptive rehabilitation are crucial for stroke patients suffering from a proprioception deficit.

A self-training/rehabilitation system for stroke survivors would be beneficial for society. The burden of rehabilitation costs on the healthcare system has a great impact on the quality of care that stroke survivors receive [49]. Large numbers of in- and outpatients compared to low numbers of therapists and caregivers have significant health-related consequences for caregivers as well as patients [50]. Self-training at home is one option that could provide relief to the healthcare system and reduce the burden on therapists and clinicians. In this study, our objective was to actively involve the user through a bimanual training protocol combined with tactile feedback to improve his motor learning and neuromotor rehabilitation. We added the Kinect to investigate its potential for a self-training/rehabilitation system and applied its joint position data for providing the participant with tactile feedback. Our goal was to develop an affordable and medically beneficial system. We hypothesized that the combined bimanual movement with tactile feedback would result in improved rehabilitation output.

## 2. Methods

A 65-year-old male left chronic stroke survivor with a stroke onset of 8 years was recruited for the study. He did not receive any rehabilitation treatment during the study, which took place at Simon Fraser University (SFU). He did not have any spasticity in his affected left limb. The Office of Research Ethics at SFU approved the clinical study protocol (No. 2012s0711), and the inclusion criteria were as follows:

- the absence of primary joint trauma and dislocation (elbow and shoulder);
- no history of fractures and peripheral nerve paralysis;
- normal cognitive score >25 with the Mini-Mental State Examination [51];
- ability to understand instructions in English;
- ability to sit on a chair and to perform tasks;
- mild to moderate paralysis (Fugl-Meyer assessment (FMA) > 19);
- more than 60 degrees of active range of motion for shoulder abduction, shoulder flexion, and elbow flexion.

### 2.1. Training Protocol

**Figure 1:**
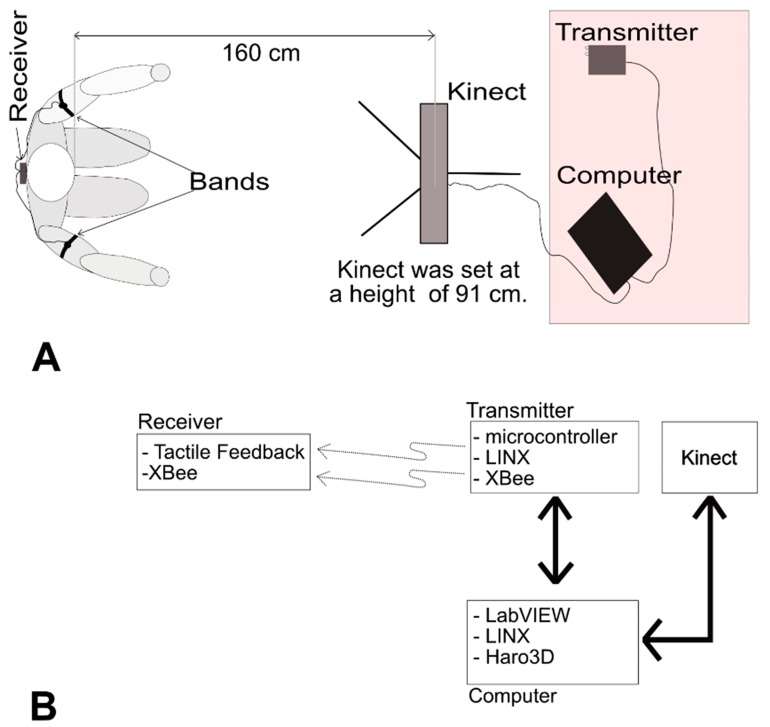
The training/rehabilitation system is shown: (**A**) It consists of a receiver with two bands that are put on the shoulders of the user for tactile feedback; a Kinect sensor placed 160 cm away from the user at a height of 91 cm; a transmitter that communicates with the receiver wirelessly, sending commands for tactile feedback, with two light emitting diodes (LEDs) serving as left- and right-arm visual feedback; and a computer connected to the Kinect and transmitter, which processes the joint information and executes a command according to the algorithm. (**B**) Hardware and software applied in the training system.

The participant came to the lab 3 times per week for 6 weeks. Two training protocols of “contralateral arm matching” and “both arms moving together” were used during the training sessions. Each of these training sessions lasted a maximum of 1 h. Two arm bands, each connected to a vibrating motor, were placed on the participant’s brachialis muscles. The two motors were connected to a receiver. The participant sat in an armchair in a comfortable position and was blindfolded (Figure 1) in order to eliminate the influence of vision on proprioception. For both training protocols, the participant carried out two tasks of “shoulder abduction” and “shoulder flexion” at angles close to 30 and 60 degrees. The two angles were both within the range of motion of the participant. The participant carried out 15 repetitions for each angle in two sets. The first set consisted of 10 repetitions and the second set included 5 repetitions for a total of 15 repetitions. The participant was allowed a short break between sets.

For example, in the “contralateral arm matching” protocol, the unaffected arm of the participant was set to an angle close to 30 degrees. Then, he was requested to keep the unaffected arm in the specified angle while trying to match the position with the affected arm. Whenever the two arms matched, a vibration was delivered to both brachialis muscles. In the “both arms moving together” protocol, the two arms were first approximately set to an angle of either 30 or 60 degrees. Then, the participant had to return to a relaxed position before moving both arms back to the remembered specified angle. Whichever arm lagged behind the other arm in resuming the specified angle received a vibration. Therefore, the participant’s goal was to move both arms to the specified angle without any lag, consequently not getting any vibration in his arms.

### 2.2. Assessment Protocol

**Figure 2:**
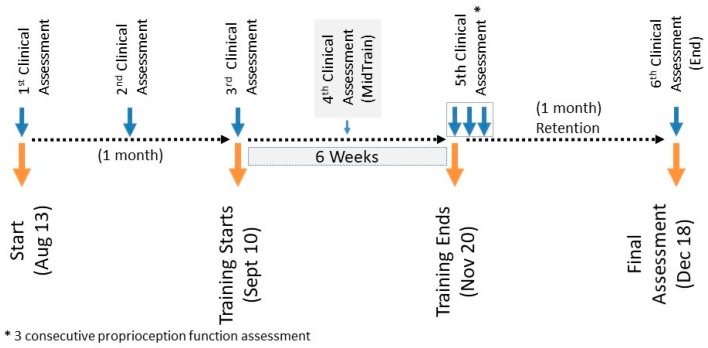
Study timeline.

Three baseline assessments with an interval of 2 weeks at the beginning of the training were made: one assessment in the middle of the training (MidTrain), one assessment at the end of the training (PostTrain), followed by another assessment one month after the training (End) (Figure 2). Multiple baseline assessments have commonly been used in other studies [52,53,54,55]. These baseline assessments were all done within a one-month period. Six clinical assessment sessions were done in total. Proprioception assessments were included in all clinical assessment sessions. An extra two sessions of the proprioceptive assessment only were performed following the 5th clinical assessment session after the training period.

**Figure 3:**
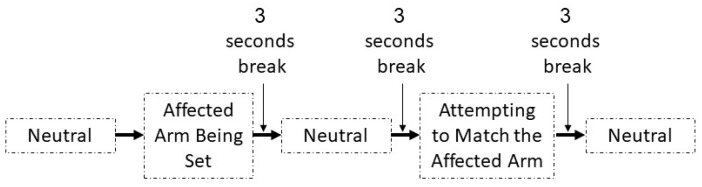
Steps in assessment of ipsilateral movements.

**Figure 4:**
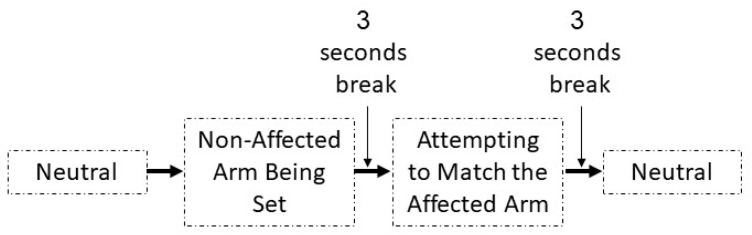
Contralateral movements assessment steps.

Two ipsilateral and contralateral arm matching tasks, each consisting of three movements (shoulder abduction, shoulder flexion, and elbow flexion), were used. Movements were performed at both 30 and 60 degree angles. For example, for the ipsilateral arm matching task (Figure 3), an experimenter positioned the affected arm of the participant at 30 degrees of shoulder abduction. The participant was requested to keep the arm in that position for ~5 s before returning to a relaxed initial position. Then, after another ~5 s delay, the participant moved the affected arm back to the remembered position. An experimenter measured this shoulder abduction angle manually using a goniometer. The same procedure was repeated for the 60 degree angle and for the other two movements. For the contralateral arm matching task (Figure 4), the procedure was the same as described above, except that the unaffected arm was moved first, and the patient had to match the angle with the affected arm.

A Fugl-Meyer assessment (FMA) [56], a Wolf motor function test (WMFT) [57], and a proprioception assessment [58] using angles measured with a goniometer were used for pre- and post-training evaluations. The FMA was applied for measuring UE and lower extremity (LE) motor and sensory impairment [56]. The FMA consists of 5 domains, which include motor, sensory, balance, range of motion, and joint pain domains. The assessment of UEs and LEs is part of the motor domain, which has a well-established reliability [59,60]. The validity of the FMA has been investigated with other scores, such as the functional independence measure, and an excellent correlation between the FMA and other scores was found [61]. The smallest real difference was considered to be 10% of the highest score [62]. The FMA is a three-point ordinal scale (0–2) and is used for the assessment of the motor and sensory FMA. Voluntary limb movement was assessed by the FMA motor assessment, which includes a UE subscale (33 items with a score range between 0 and 66) and an LE subscale (17 items with a score ranging between 0 and 34). The total motor FMA is 100 [56].

The WMFT is a time-based method for evaluating UE performance that consists of 15 tasks. The first 6 tasks consist of joint segment movements. The remaining tasks involve time-integrative functional movements [57]. The test–retest reliability of the WMFT is very high, and it has inter-rater reliability and internal consistency [63,64]. The WMFT has an excellent correlation with the action research arm test [63], suggesting it has good validity. In the literature, 1.5 to 2 s of change in the WMFT are considered to be clinically important [65].

For the proprioception assessment, a goniometer was used for pre- and post-training evaluations. Sabari et al. [66] have found a high intra-class correlation coefficient > 0.9 intra-rater reliability for active and passive measurements at the same position, with comparable measurements between trial 1 and trial 2.

## 3. System

LabVIEW and Kinect were used for both training and assessment protocols. LabVIEW was used for the real-time data collection and analysis of the data received from Kinect.

### 3.1. Receiver

**Figure 5:**
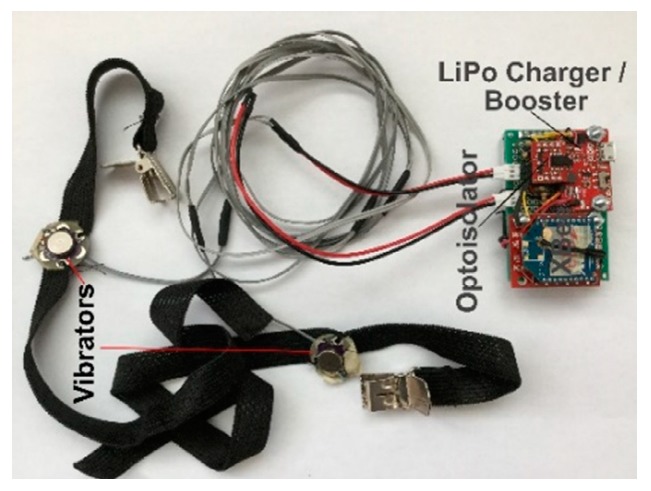
Prototype receiver.

The receiver shown in Figure 5 consists of a power source, a power regulator and charger, an XBee receiver module, an optoisolator, and two bands (each connected to a vibrating motor).

### 3.2. Transmitter

**Figure 6:**
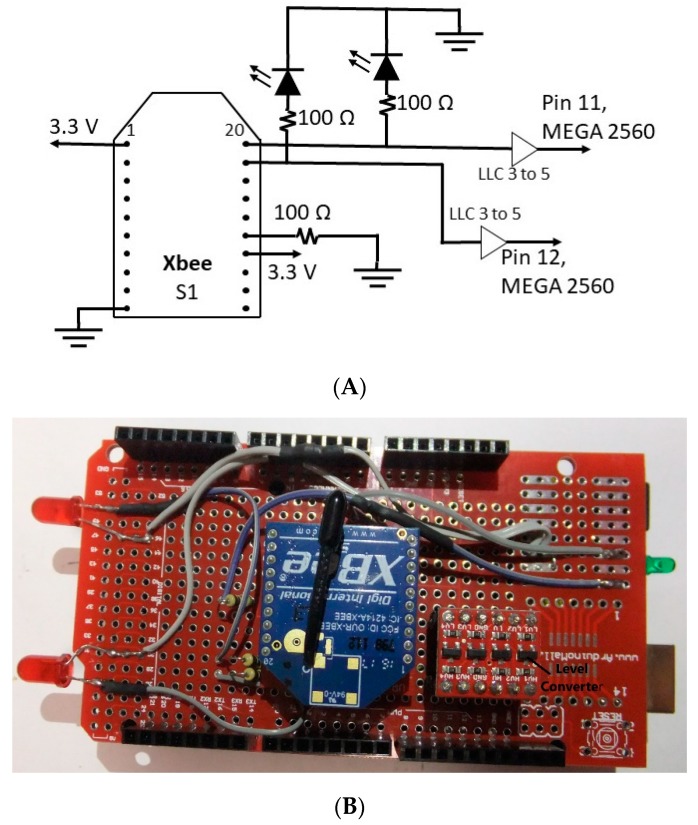
(**A**) Transmitter circuitry; (**B**) prototype transmitter. LLC 3 to 5: logic level converter 3 V to 5 V.

A transmitter connected to a personal computer was used as an interface mean between the PC and the receiver. The circuitry and the prototyped transmitter are shown in Figure 6. The transmitter consists of an Arduino MEGA 2560, a logic level converter (BOB-12009), an XBee module, and two light emitting diodes (LEDs). The LEDs are used for representing the left and right arms/forearms for visual feedback purposes.

### 3.3. Software and Hardware

#### 3.3.1. Hardware

Kinect was used to collect the user’s body joint information. The joint positions are defined in the Kinect camera space, which consists of three single *x*, *y*, and *z* positions. The origin of the camera space is the center of the depth or infrared sensor of the Kinect [67]. The joint position data are recorded by the Kinect accurately, while the joints are not occluded. However, this is not the case in terms of human movement [68,69]. The occlusion issue became even greater for some joints as the user carried out the movement while sitting.

XBee (XBee is the trademark of DIGI International, Inc.) was used for wireless communication between the receiver and transmitter. XBee is a module that allows for serial communication between devices over serial ports. “One-way of wireless communication” [70] was set up for the two XBee modules on the transmitter and receiver. XBee module settings were made by using XCTU (XCTU is a free multiplatform application provided by DIGI International, Inc.) and by using the following parameters (listed in Table 1).

#### 3.3.2. LabVIEW

**Figure 7:**
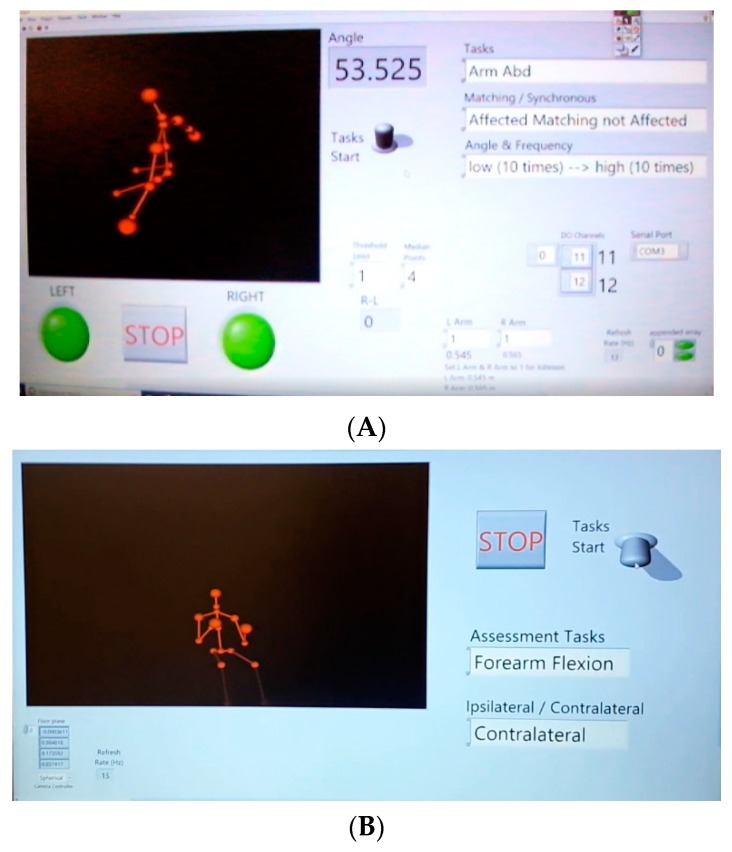
(**A**) LabVIEW training program; (**B**) LabVIEW assessment program.

LabVIEW (LabVIEW is the trademark of National Instruments) was used as the interface between the Kinect and the computer using Haro3D (Haro3D is a product of HaroTek LLC), a library allowing users access to the functionalities of Kinect. In order to interface between LabVIEW and the transmitter, LINX was used. LINX is open source and provides an easy way for LabVIEW and Arduino to interact [71]. Upon installing the hardware abstraction layer on LabVIEW and then on Arduino by using LabVIEW (Tools → MakerHub → LINX → LINX → Firmware Wizard), a single interface between LabVIEW and Arduino MEGA 2560 was made, allowing for seamless communication. In other words, the virtual instruments of LINX were installed on the PC to communicate in a serial manner with the LINX firmware on the Arduino, which allowed for the manipulation of the Arduino output. Screenshots of the training and assessment protocols are shown in Figure 7.

Two tasks of “arm abduction” and “arm flexion” were carried out by the participant. The two joints wrist (w) and shoulder (s) were chosen for upper limb movement comparison because both joints had minimal occlusion during the two movements. Both the w and s joints created two vectors, and the angle between the two line segments was defined as θ [72],

(1) $$cos\theta =\frac{w^{T}s}{\left\|w\|\|s\right\|}=u^{T}v,$$

where **u** and **v** are unit vectors and are expressed by the following formula:

$$u=\frac{1}{\left\|w\right\|}w\;\mathrm{and}\;v=\frac{1}{\left\|s\right\|}s.$$

The Euclidean distance between joints w and s was found as 72

(2) $$\|w-s\|=\sqrt{{\left(x_{w}-x_{s}\right)}^{2}+{\left(y_{w}-y_{s}\right)}^{2}+{\left(z_{w}-z_{s}\right)}^{2}},$$

where w and s stand for wrist and shoulder joint position data, respectively.

The distance between w and s was used for movement comparisons because it required a minimum amount of computations. Considering the real-time processing of the Kinect camera information, methods with a lower computational cost were preferable.

## 4. Results

**Figure 8:**
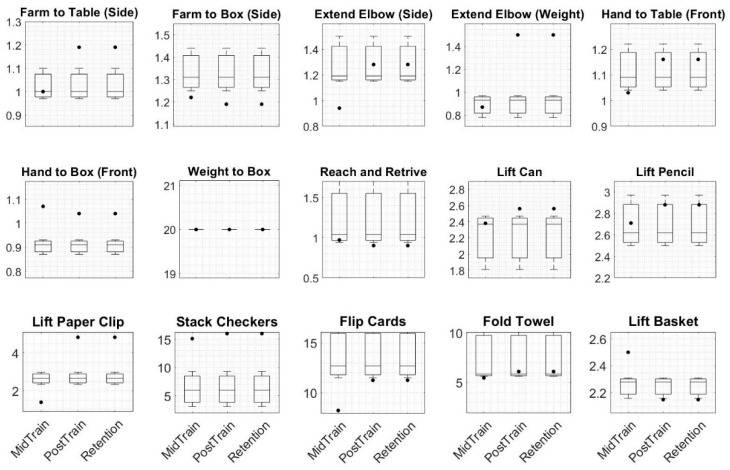
The boxplots for the Wolf motor function test (WMFT) are shown. The three baselines are plotted as boxplots in subplots. The scores for mid training, post training, and retention (one month after six weeks of training) are shown as black-filled dots and are respectively named MidTrain, PostTrain, and Retention. The *y* axis does not have any unit. The total scores of the four assessments were 65, 65, 74, and 66, respectively. Abbreviations: Farm, forearm.

**Figure 9:**
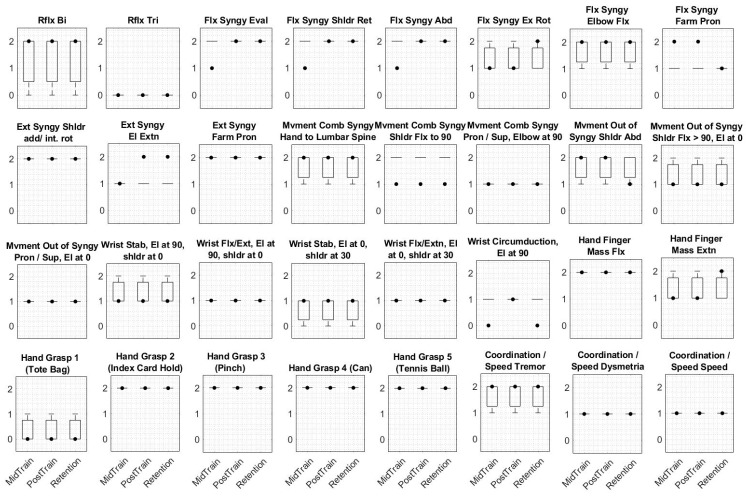
The boxplots for the Fugl-Meyer assessment (FMA) are shown. The three baselines are plotted as boxplots in subplots. The scores for mid training, post training, and retention (one month after six weeks of training) are shown as black-filled dots and are respectively named MidTrain, PostTrain, and Retention. The total scores of the four assessments were 45.33, 42, 47, and 46, respectively. The *y* axis does not have any unit. Abbreviations: Rflx, reflex; Bi: biceps; Tri, triceps; Flx, flexor; Syngy, synergy; Eval, evaluation; Shldr, shoulder; Ret, retraction; Abd, abduction; Ex, external; Rot, rotation; Pron, pronation; Ext, extensor; add, adduction; Int, internal; rot, rotation; El, elbow; Farm, forearm; Mvment, movement; Comb, combining; Sup, supination; Extn, extension; Stab, stability; 90, 90 degrees; 0, 0 degrees.

**Figure 10:**
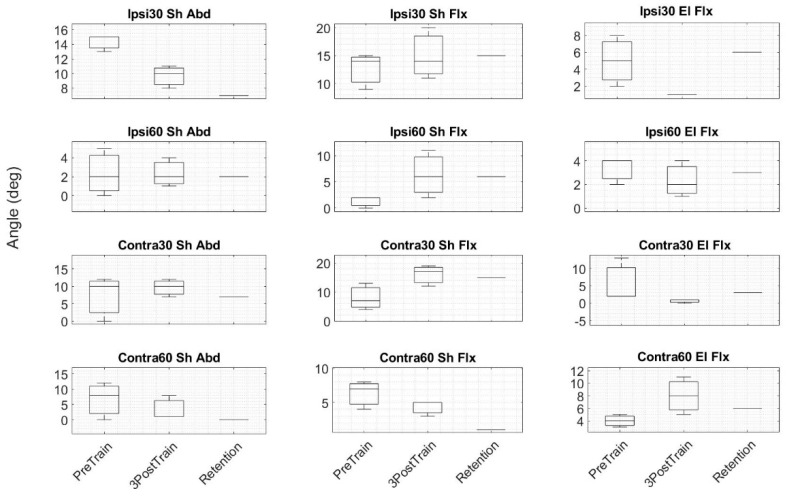
The boxplots for goniometer angle assessments of 12 tasks are shown. PreTrain: boxplot of the three baseline assessments before training starts; 3PostTrain: boxplot of the three consecutive assessments after training ends; Retention: assessment value after one month of training. Abbreviations: Ipsi, ipsilateral; Contra, contralateral; Sh, shoulder; Abd, abduction; Flx, flexion; El, elbow; 30, 30 degrees; 60, 60 degrees.

The data collected from a left hemiplegic participant based on a timeline study (shown in Figure 2) were analyzed to assess motor function (FMA and WMFT) and proprioception function (ipsilateral and contralateral arm matching angular assessments). Three assessment plots are shown in Figure 8, Figure 9 and Figure 10. The three baseline clinical assessment values used as baselines are drawn as boxplots in the subplots of the three plots of Figure 8, Figure 9 and Figure 10. The fourth, fifth, and sixth assessments are specified as MidTrain, PostTrain, and Retention. The scores of these three assessments are shown as black-filled dots. Motor function and proprioception assessment plots are discussed below.

### 4.1. Motor Function

The scores of the PostTrain and Retention assessments for the WMFT (Figure 8) showed deterioration for nine of the items compared to the baseline scores. Four items showed improvement, and two showed no differences.

Except for seven tasks that showed improvement on the Retention assessment for the FMA (Figure 9), the rest of the tasks showed almost no difference compared to the mean values of the boxplots.

### 4.2. Proprioception

Measurements for each angle (30 degrees and 60 degrees) for shoulder abduction, shoulder flexion, and elbow flexion for both “ipsilateral arm matching” and “contralateral arm matching” were collected using a goniometer. The results are shown in Figure 10. The Retention assessment scores compared to PreTrain assessment values showed improvement in four conditions (30 degrees of ipsilateral shoulder abduction, 30 degrees of contralateral shoulder abduction, 60 degrees of contralateral shoulder abduction, and 30 degrees of contralateral elbow flexion). Three pre- and post-training values were considered as two groups of independent variables. A Kruskal–Wallis H test was used for the two groups’ statistical analysis. At a 1% significance level, no significant difference was found between the two groups.

## 5. Discussion

The objective of this study was to design and develop a bimanual rehabilitation/training system combined with tactile feedback and Kinect for stroke survivors to verify the feasibility of our methodology. The participant was able to perform two types of trainings protocols (ipsilateral and contralateral arm matching) successfully by getting haptic feedback. The results showed improvement in some conditions after six weeks of training. Even for some tasks with no or less than median improvement (60 degrees of ipsilateral shoulder abduction (Ipsi60 Sh Abd) or 30 degrees of contralateral shoulder abduction (Contra30 Sh Abd), for example), the percentile angular errors were reduced, which is encouraging. Compared to 30 degrees of angular motion, 60 degrees of angular motion showed less improvement in both the median and the percentiles. Three reasons are proposed for this observation. First, the stiffness and soreness in the muscles of the shoulder affected the larger abduction and flexion movements. This was a fact that the participant himself confirmed. Second, as the traveling distance to a target increased, larger errors were produced [73]. Third, the weakness of the deltoid muscles as the result of paralysis influenced the range of motion.

During the proprioception assessments, the participant’s arm was passively positioned by an experimenter, and as a result, the time between positioning differed. The different time length between two positionings yielded different perceptions in the participant, which is called a Tau effect [74]. Herrnstadt et al. [18] have suggested that the need to memorize the location of an arm is eliminated, but the Tau effect might have been triggered, influencing the participant’s judgement. Additionally, passive arm positioning was shown to result in larger errors compared to active arm positioning [75].

Bimanual systems have been successfully applied in many clinical studies [9,10,11,12], and it has been shown that UE bimanual training is more beneficial to stroke patients compared to unilateral UE training [12]. Consistent with the meta-analysis of Chen et al. [12] on the comparison of bilateral and unilateral upper limb training in people with stroke, our results showed improvement of the stroke participant in terms of FMA total score. Although we saw improvement in the FMA total score of the participant, this may not have necessarily yielded significant improvement in functional improvement. The bimanual training concept has been combined with robotics [76,77,78,79,80,81] and with auditory cueing [82,83,84,85,86] in several studies for the rehabilitation of the UEs of stroke patients. Providing the user with physical feedback has been used in previous studies. For instance, force feedback [49] and active assisted and active resist modes for increasing cerebral activation levels [87] are examples of such feedback that has been used in studies. For our study, we embedded tactile feedback in our novel training protocol, which required active engagement of the user during the training and proved to be effective for motor improvement [88]. Including haptic feedback in our study was useful for the blindfolded participant of our study during training. He had a little trouble in knowing the location of his affected left arm. The haptic feedback assisted him to match his affected arm to the position of his unaffected arm.

The application of Kinect in our combined bimanual training with haptic feedback was novel and allowed us to verify the potential of Kinect for a self-training system for stroke survivors.

As Hackshaw [89] has suggested, conducting a well-designed small study is appropriate as long as its results are interpreted carefully, because small studies suffer from reliable estimation. In our study, we recruited one suitable stroke participant from our available list of stroke individuals. While this study helped us to confirm the feasibility of our training system, it did not firmly confirm the clinical results. A larger study will help in developing better training and assessment protocols to suit a wider range of stroke patients with mild to moderate paralysis. It will also enhance our estimation of the clinical results. For example, if we look at the FMA results of the participant, we noticed a modest improvement compared to other FMA results in the literature (e.g., Reference [90]), which made any conclusion on the significance of the clinical results difficult. The second limitation of our study was the length of the training. The participant could successfully finish six weeks of training, but we could not confirm the long-term use of the training system and the effect of tactile feedback. These two points should be addressed in future studies.

## 6. Conclusions

Rehabilitation and training are the key to recovery after stroke. Considering increasing amounts of healthcare costs, patient self-training/rehabilitation is primordial for a sustainable economy. In order to investigate the potential of an affordable and easy-to-use system for rehabilitation/training, we designed and developed a protocol and training system for stroke patients. We tested the system on a left hemiplegic participant, who was able to successfully complete six weeks of training, which showed promise in terms of system and protocol design. We also performed several functional assessments before and after the training. The proprioception assessments showed improvement in some tasks, indicating a positive effect of our protocol in rehabilitating stroke survivors.

## Figures and Tables

**Table 1:** The parameters of the two XBee modules. DL sets the 16-bit address of the receiver XBee module; MY sets the 16-bit source address for the transmitter XBee module; D0, D1, and D2 are digital inputs or outputs; IR is the sample rate.

Variables	Receiver	Transmitter
ID	3456	3456
DL	1	2
MY	2	1
D0	DO Low [4]	DI [3]
D1	DO Low [4]	DI [3]
D2	DO Low [4]	DI [3]
IR	64 (for 100 ms)	64
IT	-	1
IA	1	-
